# Eggshell Membrane/Gellan Gum Composite Hydrogels with Increased Degradability, Biocompatibility, and Anti-Swelling Properties for Effective Regeneration of Retinal Pigment Epithelium

**DOI:** 10.3390/polym12122941

**Published:** 2020-12-09

**Authors:** Jeongmin Choi, Jaewoo Lee, Myeong Eun Shin, Suyoung Been, Dae Hoon Lee, Gilson Khang

**Affiliations:** 1 Department of Bionanotechnology and Bio-Convergence Engineering, Jeonbuk National University, Jeonju 54896, Korea; cjm931@jbnu.ac.kr (J.C.); meshin@jbnu.ac.kr (M.E.S.); qlstndud@jbnu.ac.kr (S.B.); ldh7149@jbnu.ac.kr (D.H.L.); 2 Department of Polymer-Nano Science and Technology, Jeonbuk National University, Jeonju 54896, Korea

**Keywords:** tissue engineering, retinal pigment epithelium, injectable hydrogel, degradability, swelling, biocompatibility, gellan gum, eggshell membrane

## Abstract

A gellan gum (GG) hydrogel must demonstrate a number of critical qualities—low viscosity, degradability, desirable mechanical properties, anti-swelling properties, and biocompatibility—in order to be regarded as suitable for retinal pigment epithelium (RPE) regeneration. In this study, we investigated whether the application of an eggshell membrane (ESM) to a GG hydrogel improved these critical attributes. The crosslinking of the ESM/GG hydrogels was most effectively reduced, when a 4 *w*/*v*% ESM was used, leading to a 40% less viscosity and a 30% higher degradation efficiency than a pure GG hydrogel. The compressive moduli of the ESM/GG hydrogels were maintained, as the smaller pores formed by the addition of the ESM compensated for the slightly weakened mechanical properties of the ESM/GG hydrogels. Meanwhile, due to the relatively low hydrophilicity of ESM, a 4 *w*/*v*% ESM enabled an ESM/GG hydrogel to swell 30% less than a pure GG hydrogel. Finally, the similarity in components between the ESM and RPE cells facilitated the proliferation of the latter without any significant cytotoxicity.

## 1. Introduction

The numbers of elderly people in many countries have grown more than ever before, as life expectancy is increasing with the steady development of medical techniques. As a result, chronic diseases that become more prevalent with age, including cardiovascular diseases, cancer, diabetes, chronic respiratory diseases, and visual impairments, have emerged as social concerns. Among these, visual impairment has attracted a great deal of attention from researchers, as the number of patients suffering from visual impairment has skyrocketed over the past two decades [1]. Today, it is common knowledge among public health practitioners that visual impairment among the elderly will have social costs, including but not limited to increased public health expenditures to directly remediate cases. This has prompted the research community to explore diverse means of correcting visual impairment-related diseases, including surgery [2,3] and cell therapy [4,5].

In considering the causes and treatment of visual impairment, special attention must be paid to the retinal pigment epithelium (RPE). RPE cells assume a diverse range of physiological functions, such as absorbing stray light [5], transporting materials [6], acting as a protective barrier [7], phagocytosing materials [7], and regulating immunities [8]. By virtue of their essential role, their degeneration gives rise to serious visual dysfunction. Indeed, RPE loss is highly correlated with age-related macular degeneration (AMD), itself the primary cause of severe visual impairments (loss of central vision or even blindness) [5,9,10]. Moreover, as degenerative retinal diseases are likely to result in complications such as sub-retinal choroidal neovascularization [11], necessary and appropriate actions should be taken before it is too late to prevent severe and irreversible damage accompanied by a lifelong visual disability.

In 2015, Lanza et al. [12] undertook a groundbreaking effort to treat RPE-associated diseases by transplanting RPE cells differentiated from human embryonic stem cells (hESC) into patients with AMD and Stargardt’s macular dystrophy. However, although hESC-derived cells could safely provide a novel source of cells to cover various unmet medical conditions arising from RPE degeneration, there are constraints imposed by retinal transfer of hESC-RPE using a suspension method such as the difficulty in correctly delivering it to a movable treatment region [13]. As a result, there is still considerable demand to look for an alternative remedy.

Among hydrogel drug delivery systems, injectable hydrogels have earned a reputation as attractive matrices for delivering drugs in a controlled and targeted manner [14]. Hydrogels can promote cell migration, as their matrix can be penetrated with water molecules and swollen, and then cells are released to the target site [15,16,17]. RPE cell transplantation using a hydrogel as a carrier offers an accessible alternative to retinal regeneration, taking into account that scaffold-based transplantation is more effective in obtaining living cells than the alternative suspension method [18]. Hydrogels are particularly suitable for retinal transplantation, as they are high in moisture, highly porous, exceptionally biocompatible and are similar in their physical structure and chemical composition to the native extracellular matrix [19,20,21]. A number of natural and synthetic biomaterials have been adapted for scaffold manufacture using various fabrication techniques to create 3D environments that mimic an extracellular matrix [22]. The hydrogel gellan gum (GG) was chosen for this study for its shape-retaining abilities, along with its excellent chemical, thermal, and enzymatic stabilities [4,23,24]. Despite a large number of advantages of GG, there is still room for further improvement of its degradability and injectability.

To improve those two characteristics, merely lowering the concentration of crosslinker used to form GG hydrogels may appear enough. Unfortunately, it is challenging to achieve superior degradability and injectability in this way while maintaining their morphologies. A low amount of crosslinker can cause greater swelling in water, making it hard for GG hydrogels to keep their shapes to prevent a sharp increase in the intraocular pressure during cell delivery in the body [25,26,27]. In this paper, we demonstrate that eggshell membranes (ESMs) extracted from natural eggshells have untapped potential to enhance GG hydrogels’ degradability and biocompatibility while reducing scaffold swelling. An ESM consisting of 10%–20% collagen [28], 0.5%–4.5% hyaluronic acid [28,29], and 0.5%–1.7% sialic acid [30] was hypothesized to improve these functions without changing the crosslinker concentration in the light of the fact that it is a degradable and biocompatible material less relevant to swelling. The ESM was confirmed to be well-suited for maintaining the balance between gradual degradation and preservation of proper morphology, thereby enabling the creation of GG hydrogels with improved degradability, biocompatibility, and anti-swelling properties.

## 2. Materials and Methods

### 2.1. Preparation of ESM/GG Scaffolds

Unless otherwise noted, all chemicals and materials were used as received from Sigma-Aldrich, except for eggs obtained from a local market (Jeonju, Korea) to separate ESMs. Raw chicken eggs were used to extract ESM. Eggshells were washed with a 0.05 M sodium carbonate solution, and ESMs were peeled off from the washed eggshells. The collected ESMs were freeze-dried and subsequently cryo-ground to a powder. The powder was sieved with a 100 µm mesh screen to make them uniform in size. The sieved ESM powder was added to aqueous GG solutions consisting of 2 *w*/*v*% GG and 0.03 *w*/*v*% calcium chloride, which was used as a crosslinking agent, at final concentrations of 0, 1, 2, 3, and 4 *w*/*v*% at 70 °C (Table 1). Note that a 5 *w*/*v*% sample was ruled out in this study, because it was too mushy. After confirming that the powder was dispersed in the GG solutions, gelation was performed by lowering the temperature from 70 °C to room temperature to acquire a cylindrical scaffold with a diameter of 6 mm and a height of 4 mm. The as-prepared GG hydrogels containing 0, 1, 2, 3, and 4 *w*/*v*% ESM were marked as E0, E1, E2, E3, and E4, respectively.

### 2.2. Characterizations of ESM/GG Solutions and Hydrogels

#### 2.2.1. Rheological Analysis

The viscosities of the aqueous ESM/GG solutions (5 mL) containing crosslinkers were measured while inducing the liquid-to-gel transition of the solutions using a viscometer (DV1, AMETEK Brookfield, Middleborough, MA, USA) by ramping down the temperature (1 °C every minute) from 70 to 30 °C at a rotor speed of 0.6 rpm. The viscosity curve was recorded only until the gelation was finished, although the temperature did not reach the lowest set temperature (i.e., 30 °C). Accordingly, the measurement of each viscosity was ceased at each gelation temperature.

#### 2.2.2. Compressive Moduli of ESM/GG Hydrogels

To determine how the mechanical properties of the hydrogels varied with the ESM content, the compressive strengths of the wet ESM/GG hydrogels were tested using a Texture Analyzer (TMS-Pro, Food Technology Corporation, Sterling, VA, USA) equipped with a 50 N load cell. The radii and heights of all of the cylindrical specimens were 3 and 4 mm, respectively. Compressive strength measurement was performed, until the compressive strength reached 10 N at a compression rate of 1 mm min^−1^. Afterward, the obtained compressive strength measurement was translated into the compressive modulus based on the Hooke’s law:

E = σ/ε,(1)

where E is the compressive modulus, σ is the applied compressive stress, and ε is the strain (compressed length/original length).

#### 2.2.3. Morphologies and Porosities of ESM/GG Hydrogels

ESM/GG hydrogel morphologies were observed using a Bio-LV scanning electron microscope (SEM, S-3000N, HITACHI, Tokyo, Japan). Prior to observation, the ESM/GG hydrogels were prepared, lyophilized, cut into cross sections and coated with platinum in a sputter coater (SC7640, Quorumtech, Lewes, UK).

The porosities of the ESM/GG hydrogels were estimated as reported previously [31]. First, the ESM/GG hydrogels were lyophilized. Next, the initial volume of water used for the porosity measurement was measured and marked as V_1_. Freeze-dried hydrogels were then immersed in the water and allowed to stand for 10 min to fully fill the pores inside the hydrogels with water. The volume of the water, including the lyophilized hydrogels, was measured and marked as V_2_. Finally, the immersed hydrogels were removed from water, which was accompanied by a certain amount of water loss due to the hydrogels’ water uptake. The final volume of water was measured after the removal of the soaked hydrogels and marked as V_3_. The porosities of the ESM/GG hydrogels were determined using the following relationship:

(2) $$\mathrm{Porosity}\;\left(\%\right)\;=\;\frac{V_{1}{\;-\;V}_{3}}{V_{2}\;{-\;V}_{3}}\times 100.$$

#### 2.2.4. Analyses for the Sol Fraction, Swelling, and Disintegration of ESM/GG Hydrogels

The sol fractions of the ESM/GG hydrogels were evaluated by comparing their masses before and after their storage in distilled water for 1 h. Every ESM/GG hydrogel was lyophilized to measure its initial mass (W_i_). Hydrogels were left in distilled water for approximately 1 h under stirring conditions to allow the remaining sol to be dissolved and removed from the hydrogels. Afterward, the sol-free hydrogels were freeze-dried to measure their final weights, which did not include any sol (W_g_). Sol fraction was estimated based on the following relationship [32]:

(3) $$\mathrm{Sol}\;\mathrm{fraction}\;\left(\%\right)=\;\frac{W_{i}{\;-\;W}_{g}}{W_{i}}\times 100.$$

Meanwhile, the swelling ratios of the ESM/GG hydrogels were assessed by contrasting the masses (W_i_) of the freeze-dried hydrogels with the masses (W_s_) of swollen hydrogels after immersion in distilled water. Swelling ratio was calculated using the following relationship [32]:

(4) $$\mathrm{Swelling}\;\mathrm{ratio}\;\left(\%\right)\;=\;\frac{W_{s}{\;-\;W}_{i}}{W_{i}}\times 100.$$

Lastly, the degradation degrees of the ESM/GG hydrogels were investigated by measuring the ratio of the masses of the hydrogels before and after exposure to a phosphate-buffered saline (PBS) solution (pH 7.4) at 37 °C. The lyophilized hydrogel’s initial mass was marked as W_i_, while the final mass of the hydrogel partially disintegrated in a PBS solution was marked as W_d_. Weight loss ratio was determined using the following equation [33]:

(5) $$\mathrm{Weight}\;\mathrm{loss}\;\mathrm{ratio}\;\left(\%\right)=\;\frac{W_{i}{\;-\;W}_{d}}{W_{i}}\times 100.$$

### 2.3. In Vitro Tests

#### 2.3.1. RPE Cell Isolation and Cell Culture

All the animal experiments for this study were carried out with the approval of the Jeonbuk National University Animal Care Committee, Jeonju, Republic of Korea (JBNU 2016-50). All processes abided by the guidelines set by the Jeonbuk National University Animal Care Committee and were done in a manner to minimize animal suffering. Six-week-old colored rabbits (Pigmented Rabbit, KOATECH, Pyeongtaek, Korea) were used for cell separation. Dulbecco’s Modified Eagle Medium/F-12 (Gibco, Waltham, MA, USA) with 10% fetal bovine serum (Gibco, Waltham, MA, USA) and 1% Antibiotic-Antimycic (Gibco, Waltham, MA, USA) was used in cell culture. The temperature was 37 °C, and the carbon dioxide concentration for cultivation was 5%. RPE cells were isolated from colored rabbit eyes. The isolated RPE cells were digested with 2% collagenase A (Roche, Penzberg, Germany) for 1 h and then transferred to fresh cell culture dishes. The medium was replaced every 2 days and washed every 5 days with an Antibiotic-Antimycotic. Two postcolumn cells (cell passage number 2, P2) were used in this study.

#### 2.3.2. Cell Encapsulation in ESM/GG Hydrogels

The cells were separated from the cell culture dishes with a cell scraper. The separated cells were added to an aqueous ESM/GG solution at a density of 5 × 10^6^ cells mL^−1^, followed by gelation.

#### 2.3.3. Cell Viability and Proliferation Assay

Cell viability and proliferation were analyzed by Thiazolyl blue tetrazolium bromide (MTT) and Live/Dead assays. The MTT assay was performed every seven days after cell encapsulation, i.e., on days 1, 7, 14, 21, and 28. Thiazolyl blue tetrazolium bromide (Alfa Aesar, Haverhill, MA, USA) was dissolved in PBS at a concentration of 5 mg mL^−1^ to prepare an MTT solution. The hydrogels were added to 1 mL of a fresh medium. Afterward, 0.1 mL of the MTT solution was added to the medium, followed by a 4 h incubation at 36.5 °C. After incubation, the hydrogels were placed in 1 mL of DMSO to dissolve purple formazan formed by the reduction of MTT and shaken for 30 min to mix, at which point 0.2 mL of DMSO including purple formazan was transferred to 96 wells to measure the absorbance at 570 nm.

The Live/Dead assay was conducted every seven days after cell encapsulation, i.e., on days 1, 7, and 14. A Live/Dead cell imaging kit (Invitrogen, Carlsbad, CA, USA) was used to observe the distribution of live and dead cells with a confocal laser scanning microscope (CLSM; LSM 880 with Airyscan, Carl Zeiss, Oberkochen, Germany) after fluorescent staining.

#### 2.3.4. Gene Expression Assay

Expressed proteins were confirmed by RT-PCR. Cells were extracted from the hydrogels using a glass tissue grinder and lysed using 1 mL of Trizol (Invitrogen, Carlsbad, CA, USA). Subsequently, 0.2 mL of chloroform was added to the lysed cells, and the cell debris was centrifuged at 12,000× *g* for 15 min at 4 °C to collect RNA from the supernatant fraction. The collected RNA was precipitated with isopropanol and spun down as described above. The RNA pellet was washed with ethanol with the same method as the above centrifugation procedure. An RNA solution was then prepared by dissolving the RNA in 40 µL of RNase-free distilled water (Invitrogen, Carlsbad, CA, USA). After determining the concentration of the resulting RNA solution, the required amount of RNA was added to a One-Step RT-PCR Kit (Enzynomics, Daejeon, Korea) tube along with expression markers such as β-actin, IL1B, Col I, Col II, MITF, NPR-A, Rhodopsin, and RPE65. Afterward, RT-PCR was performed with a TAKARA PCR Thermal Cycler Dice (TaKaRa, Kyoto, Japan). Finally, electrophoresis was performed to analyze the PCR products on a 1% agarose gel by means of Mupid-One (TaKaRa, Kyoto, Japan) for 30 min at 100 V. The electrophoresed PCR products were visualized by a UV transilluminator (MultiImage Light Cabinet, Alpha Innotech, San Leandro, CA, USA).

### 2.4. Statistical Analysis

GraphPad Prism 5.0 software (La Jolla, San Diego County, CA, USA) was used for statistical analysis. The resulting values were represented by means and standard deviations. *p* values were analyzed through the one-way ANOVA test and specified as follows: NS (not significant) *p* > 0.05, * *p* < 0.05, ** *p* < 0.01, and *** *p* < 0.001.

## 3. Results and Discussion

### 3.1. Correlation between the ESM Addition and Injectability

**Figure 1:**
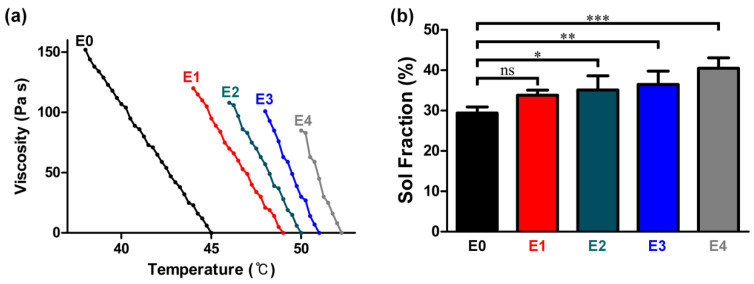
(**a**) Viscosities of ESM/GG hydrogel solutions including different concentrations of ESMs at different temperatures. (**b**) Sol fractions of ESM/GG hydrogels containing different concentrations of ESMs. Error bars mean the standard deviation (n = 4). One-way ANOVA with Dunnett’s multiple comparison test was carried out to compare the sol fractions of ESM/GG hydrogels containing different concentrations of ESMs. In Graph Pad Prism 6 software, a significant difference is indicated as follows: NS (not significant) *p* > 0.05, * *p* < 0.05, ** *p* < 0.01, and *** *p* < 0.001.

Injectable hydrogels should have: (i) low viscosity, (ii) degradability, (iii) desirable mechanical properties to endure possible deformation arising from ocular movement, (iv) structural stability (anti-swelling properties), and (v) biocompatibility [34]. In this section, we examine how the ESM lowers GG hydrogel solutions’ viscosity and improves their injectability. As shown in Figure 1a, the viscosity of aqueous GG solutions containing 0, 1, 2, 3, and 4 *w*/*v*% ESM began to dramatically increase after a certain temperature threshold, which means that each GG solution began gelling at that particular temperature. Notably, the gelation temperature increased with the ESM content (i.e., 38 °C for E0, 44 °C for E1, 46 °C for E2, 48 °C for E3, and 50 °C for E4). This result may be explained by the fact that a higher gelation temperature is associated with more crosslinking junctions [35]. As glycoprotein, which is one of the crucial components of ESM, possesses carboxyl groups [36], increased ESM likely increased the absolute number of crosslinking junctions, thereby raising the gelation temperature.

**Figure 2:**
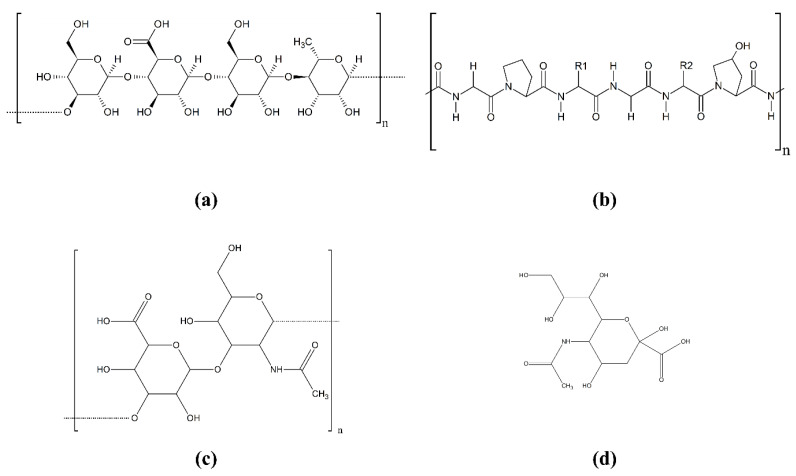
Chemical structures of GG (**a**), collagen type I (**b**), hyaluronic acid (**c**), and sialic acid (**d**). The chemical structure of collagen type I was represented as the most prevalent major component of an ESM [37,38,39,40].

**Figure 3:**
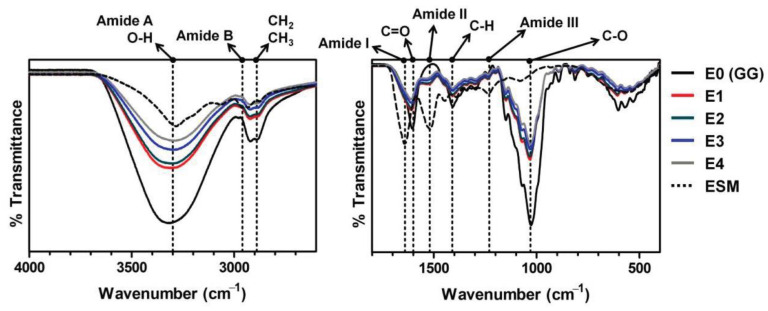
FT-IR spectra of the ESM/GG hydrogels with different amounts of ESM.

More importantly, the viscosity at the gelation temperature plummeted from 151 to 93 Pa s, as the ESM content increased from 0 to 4 *w*/*v*%. The low viscosity may be attributable to the gelation temperature being raised by the ESM, in that crosslinking could be terminated before the viscosity went higher as the gelation temperature dropped. This phenomenon was more pronounced, as ESM content increased. As such, the rapid crosslinking promoted by the added ESM is presumed to result in a relatively low crosslinking density. In addition, the low viscosity may be explained by the possibility that the crosslinking density per unit mass of ESM/GG hydrogels may drop as the ESM content increases, as the number of carboxyl groups per unit mass of ESM may be lower than those of GG (Figure 2 and Figure 3). The evidence for this second explanation can be found in the sol fraction of ESM/GG hydrogels. Specifically, Figure 1b reveals that the sol fraction of ESM/GG hydrogels increased from 29.4% to 40.5% as ESM content increased, supporting the idea that the crosslinking density of the hydrogels was reduced due to ESM loading. As such, the ESM is confirmed to decrease viscosity, which is a critical criterion for the development of improved injectable ESM/GG hydrogels.

### 3.2. Correlation between the ESM Addition and Degradability

**Figure 4:**
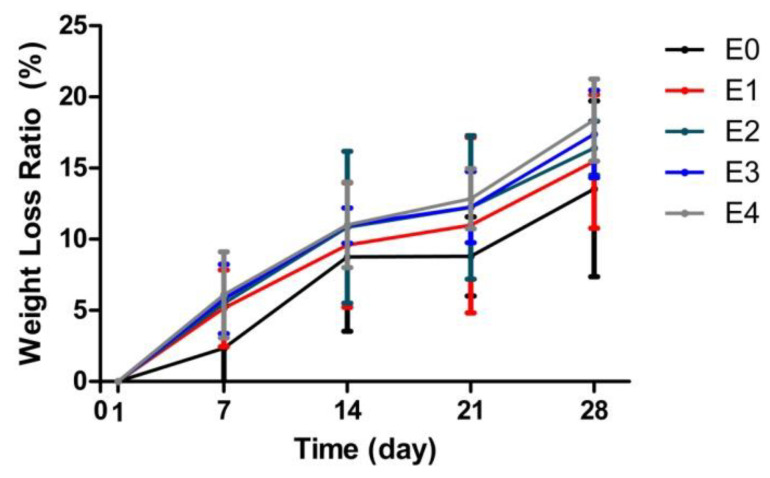
Weight loss ratios of ESM/GG hydrogels with different concentrations of ESMs to evaluate their degradability. Error bars mean the standard deviation (n = 4).

Hydrogel degradability is another critical parameter, as the transfer efficiency of cells to a target area is highly linked to the degradability of the cell carriers. We determined that the degradability of ESM/GG hydrogels was slightly improved with the loading of ESM (Figure 4), and this tendency was consistently observed over 28 days of testing. E4, in particular, represented approximately a 30% higher degradation rate than E0, taking account of their slopes (0.49 for E0 and 0.64 for E4), suggesting that the ESM will likely improve cell carrier degradability and boost cell transfer efficiency. These improvements in the degradability of ESM/GG hydrogels are also assumed to be attributable to the lower crosslinking density stemming from the ESM addition.

### 3.3. Correlation between the ESM Addition and Mechanical Properties

**Figure 5:**
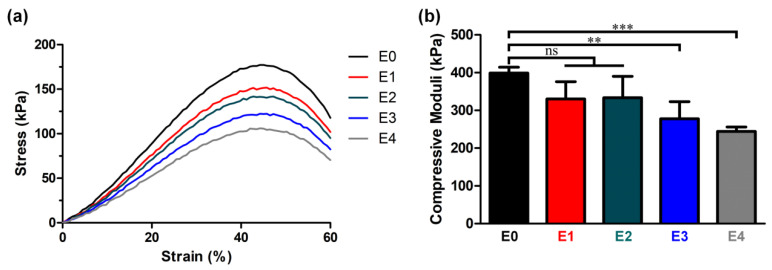
(**a**) Stress-strain curves of ESM/GG hydrogels. (**b**) Compressive moduli of ESM/GG hydrogels containing different amounts of ESMs. Error bars mean the standard deviation (n = 4). One-way ANOVA with Dunnett’s multiple comparison test was carried out to compare the compressive moduli of ESM/GG hydrogels containing different concentrations of ESMs. In Graph Pad Prism 6 software, a significant difference is indicated as follows: NS (not significant) *p* > 0.05, ** *p* < 0.01, and *** *p* < 0.001.

Safely retaining cells in the matrix until their release into the target region requires an ability to maintain the mechanical stability of the cell carriers. Injectable hydrogels must, therefore, possess appropriate mechanical properties at a level as high as RPE cells, such that they can withstand repetitive deformations that may occur during intraocular movement. Recalling that the compressive modulus of the choroid-RPE cells is approximately 4.8–6.2 kPa [31,41], it is desirable that the compressive modulus of injectable hydrogels falls into a higher, or at least a similar, range. With this in mind, it is noteworthy that the compressive moduli of all the ESM/GG hydrogels fell into a range of 244 and 399 kPa (Figure 5), although this was gradually reduced as the ESM content increased.

**Figure 6:**
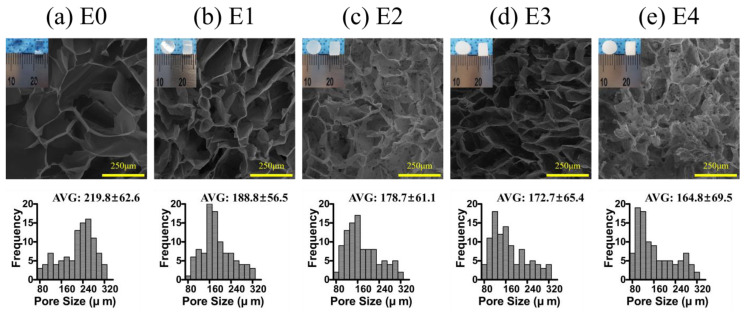
Cross-sectional SEM images and histograms representing the pore size distributions of E0 (**a**), E1 (**b**), E2 (**c**), E3 (**d**), and E4 (**e**). One hundred measurements were made to provide the pore size distribution for each sample.

**Figure 7:**
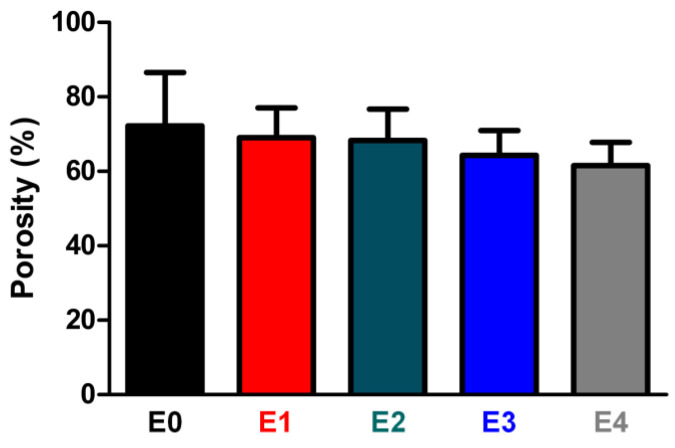
Porosities of ESM/GG hydrogels containing different amounts of ESMs. Error bars mean the standard deviation (n = 4).

The ESM likely reduced the degree of crosslinking in the ESM/GG hydrogels due to its smaller number of functional groups per unit mass than that of GG. In addition, ESM could disrupt the GG crosslinking. For those reasons, the addition of the ESM may have weakened the mechanical properties of the ESM/GG polymer composite by providing stress convergence points [42,43]. On the other hand, adding ESM to a GG solution was highly likely to entail more nucleation points, resulting in pore size and porosity reduction [44]. Indeed, we observed that pore size (Figure 6) and porosity (Figure 7) were smaller as more ESM was added. This decrease is anticipated to improve the weakened mechanical properties, as smaller pores tend to be more suitable than larger ones for enhancing the mechanical properties of a porous polymer matrix by whittling down the number of macrovoids where mechanical failure can occur [45,46]. As such, although the ESM may have appeared to have weakened the hydrogel’s mechanical properties, the detriments were not serious since the smaller pores resulting from the addition of the ESM counteracted the weakness of the ESM.

### 3.4. Correlation between the ESM Addition and Anti-Swelling Properties

**Figure 8:**
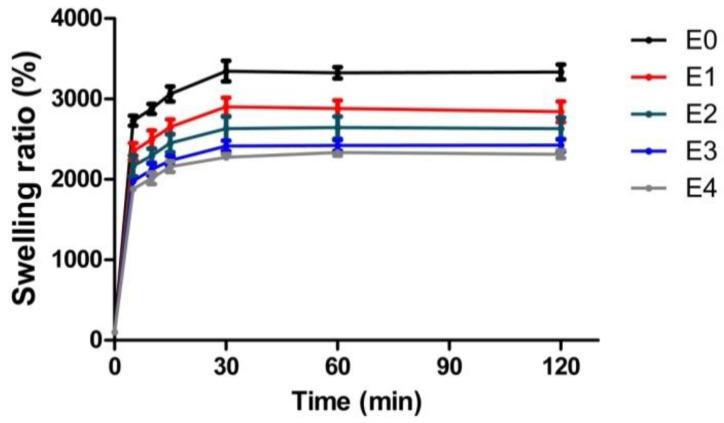
Swelling ratios of the as-prepared ESM/GG hydrogels containing various ESM amounts at different time intervals. Error bars mean the standard deviation (n = 4).

Injectable hydrogels must also satisfy the anti-swelling requirement to ensure that cell carriers do not raise intraocular pressure too high [26,27]. As hydrogel swelling correlates strongly with the amount of hydrophilic functional groups capable of absorbing water [34], we expected that the ESM addition, which has fewer hydrophilic groups than GG (Figure 2), would lower the swelling degree of ESM/GG hydrogels (Figure 8). Testing revealed that, as predicted, increased ESM resulted in a reduction in swelling in ESM/GG hydrogels.

Each of the ESM/GG hydrogels showed significant swelling 5 min after they were immersed in distilled water, regardless of the amount of ESM added (Figure 8). It is noteworthy; however, that swelling dropped from approximately 27 to 19 times as the ESM content increased from 0 to 4 *w*/*v*%. Moreover, swelling in E4 remained 30% lower than E0 throughout the 2 h test. This reduction in the swelling degree was considered comparable to those of the previously developed hydrogels (e.g., Lee et al. showed about a 15% decrease in the swelling degree [47]). Based on this observation, we concluded that the ESM decreases swelling in hydrogels owing to its relatively lower amount of hydrophilic functional groups, as compared to GG. Such anti-swelling properties of the ESM would keep the intraocular pressure low.

### 3.5. Correlation between the ESM Addition and Biocompatibility

Although the reduced hydrophilicity of the ESM had advantageous anti-swelling properties, we considered the possibility that it might negatively affect the hydrogel’s biocompatibility. With this in mind, we undertook a thorough investigation of the effect of the added ESM on biocompatibility, focusing on the following two factors: (i) cell proliferation and cytotoxicity and (ii) mRNA expression.

**Figure 9:**
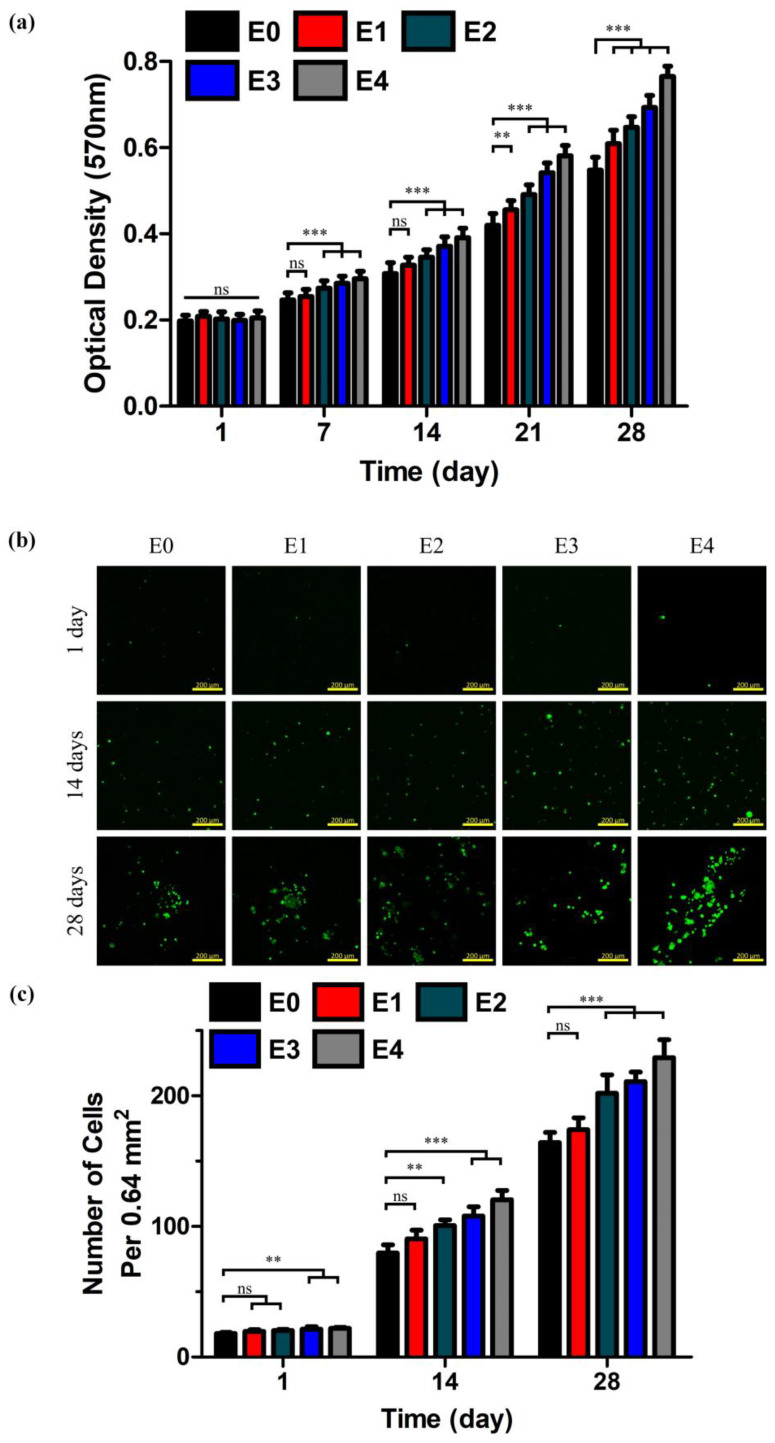
(**a**) Optical densities of formazan crystals measured at 570 nm in order to determine cell proliferation of pigment epithelium (RPE) cells cultured in the ESM/GG hydrogels based on a tetrazolium bromide (MTT) assay. CLSM images (**b**) and the number (**c**) of living cells cultured for 1, 14, and 28 days in the ESM/GG hydrogels containing different amounts of ESMs. *p* values were analyzed through the one-way ANOVA test and specified as follows: NS *p* < 0.05, ** *p* < 0.01, and *** *p* < 0.001. Error bars mean the standard deviation (n = 12 for the optical densities of formazan crystals and n = 4 for the number of living cells).

Cell proliferation and cytotoxicity were quantitatively examined by MTT assay and Live/Dead cell imaging. An MTT assay was carried out every seven days (days 1, 7, 14, 21, and 28) after seeding RPE cells at the density of 5 × 10^6^ cells mL^−1^ to determine the influence of ESM on the rate of cell proliferation. As shown in Figure 9a, there was no apparent difference in cell proliferation rate in the first MTT assay, but the rate difference became more pronounced by the day 7 assay. Most notably, E4 demonstrated approximately a 40% higher cell proliferation rate than E0 since the day 14 assay, implying that the ESM had a somewhat favorable effect on cell proliferation [48]. This improvement was quite significant, considering that the previous study showed only about a 10% increase in the proliferation rate [47]. Cytotoxicity was contrary to the trend of cell proliferation. Our testing showed that a greater number of living cells (green dots) were observed in the ESM/GG hydrogels with a higher ESM content (e.g., E4 retained 40–50% more living cells than E0 since at the time of the day 14 culture; Figure 9b,c). Overall, the ESM addition promoted cell proliferation, and it was highly unlikely that the ESM was cytotoxic.

**Figure 10:**
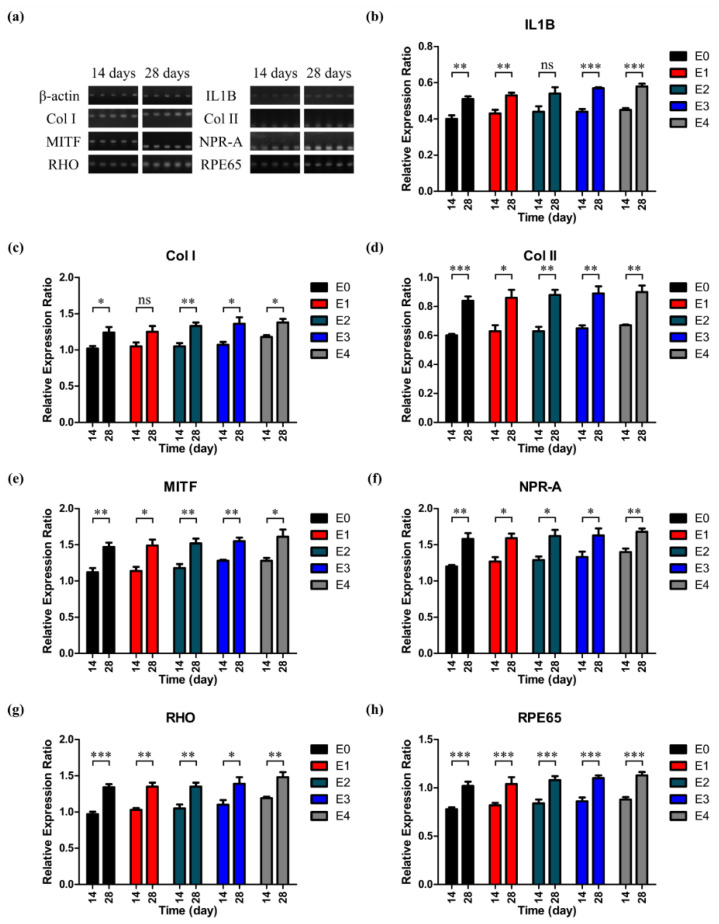
(**a**) The agarose gel images obtained after the electrophoresis of the RT-PCR products. Relative expression ratios of IL1B (**b**), Col I (**c**), Col II (**d**), MITF (**e**), NPR-A (**f**), Rhodopsin (**g**), and RPE65 (**h**) normalized based on the expression of β-actin. *p* values were analyzed through the one-way ANOVA test and specified as follows: NS (not significant) *p* > 0.05, * *p* < 0.05, ** *p* < 0.01, and *** *p* < 0.001. Error bars mean the standard deviation (n = 4).

To evaluate the influence of the ESM on the mRNA expression, we then explored how the expression of mRNAs associated with RPE varied as the amount of ESM was increased. Seven RPE-related proteins were used in the RT-PCR, and the gene expression levels were normalized based on the expression of β-actin. The details of the seven RPE-related genes are listed in Table 2. According to the gene expression levels of the seven RPE-related genes (Figure 10), all genes tested in this study were expressed at a similar level to one another, independent of the amount of ESM. There was no significant difference in the relative expression ratio between the day 14 and day 28 cultures of the as-prepared ESM/GG hydrogels with different amounts of ESMs. It is clear from these in vitro tests that the addition of ESM did not negatively affect RPE metabolism.

We focused on the following two factors to determine the root cause of how the biocompatibility of ESM/GG composites was greater than or similar to a hydrogel consisting of only GG despite the less hydrophilicity of ESM. First, while the ESM has relatively low hydrophilicity, it is mostly composed of natural fibrous proteins, such as type I collagen (Col I), which is a major component of RPE cells [54,55] and is favorable for cell proliferation [56,57]. This similarity in components between the ESM and RPE cells is deemed a positive factor for the biocompatibility of ESM/GG hydrogels. Second, as others have reported, the smaller pores induced by the ESM addition are more suitable for cell adhesion and migration [44], enabling the ESM to bring more benefits than merely offsetting the low hydrophilicity of the ESM.

## 4. Conclusions

This paper demonstrated that the ESM has significant potential to improve the degradability and anti-swelling properties of the GG hydrogels used for RPE regeneration. Furthermore, the ESM improved biocompatibility due to the similarity between the components of the ESM and RPE cells. Our testing revealed the following:

(1) ESM reduced the composite hydrogel’s crosslinking density at a reasonable level by facilitating crosslinking termination, enhancing the hydrogel solution’s injectability by lowering its viscosity.
(2) The ESM/GG hydrogel’s low crosslinking density also encouraged the hydrogel’s degradation.
(3) The increase in nucleation points formed by the ESM addition decreased the hydrogels’ pore size and porosity, partly offsetting the decrease in the mechanical properties of the hydrogel while maintaining a favorable environment for cell proliferation.
(4) The relatively low hydrophilicity of the ESM contributed to less swelling in hydrogels.
(5) The similarity in the components of the ESM and RPE cells facilitated the proliferation of RPE cells without significant cytotoxicity.
(6) These improvements were most prominent, when a 4 *w*/*v*% ESM was added to a GG solution.

## Figures and Tables

**Table 1:** The compositions (*w*/*v*%) of the mixtures used to prepare five types of eggshell membrane (ESM)/ gellan gum (GG) hydrogels.

	GG	CaCl_2_	ESM
E0	2	0.03	0
E1	2	0.03	1
E2	2	0.03	2
E3	2	0.03	3
E4	2	0.03	4

**Table 2:** Seven RPE-related genes used in the RT-PCR.

Genes	Functions or Definitions	References
IL1B	Cytokine protein that is an important mediator of the inflammatory response	[49]
Col I	Protein found in the extracellular matrices produced by RPE cells and Bruch’s membrane	[31]
Col II	Protein found in the extracellular matrices produced by RPE cells and Bruch’s membrane	[31]
MITF	Regulation of the expression of retinaldehyde binding protein 1 (Rlbp1) and retinal dehydrogenase 5 (Rdh5)	[50]
NPR-A	Regulation of the gene expression related to RPE cell proliferation or sub-retinal fluid absorption	[51]
Rhodopsin	Light-sensitive receptor protein of rod cell in the photoreceptor layer	[52]
RPE65	Activation of photoreceptor optical pigments for photon absorption and vision maintenance	[53]
